# Reducing social biases in text-based emotion prediction using semantic blinding and semantic propagation graph neural networks

**DOI:** 10.1038/s41598-026-46749-7

**Published:** 2026-05-08

**Authors:** Hubert Plisiecki

**Affiliations:** 1 Polish Academy of Sciences, Warsaw, Poland; 2 Stowarzyszenie na rzecz Otwartej Nauki (Society for Open Science), Warsaw, Poland

**Keywords:** Bias mitigation, Emotion prediction, Sentiment analysis, Graph neural networks, AI fairness, Explainable AI, Human behaviour, Computational science

## Abstract

This paper introduces the Semantic Propagation Graph Neural Network (SProp GNN), a machine learning emotion prediction (EP) architecture that relies exclusively on syntactic structures and word-level emotional cues to predict emotions in text. By semantically blinding the model to information about specific words, it is robust to social biases such as political or gender bias that have been plaguing previous machine learning-based EP systems. The SProp GNN shows performance superior to lexicon-based alternatives such as VADER (Valence Aware Dictionary and Sentiment Reasoner) and Emotlas on two different prediction tasks, and across two languages. Additionally, it approaches the accuracy of transformer-based models – with an average of 5.7% difference in accuracy scores on discrete English benchmarks, and from 0.075 to 0.145 correlation difference on English and Polish dimensional emotion prediction datasets - while significantly reducing bias in emotion prediction tasks. By offering improved explainability and reducing bias, the SProp GNN bridges the methodological gap between interpretable lexicon approaches and powerful, yet often opaque, deep learning models, offering a robust tool for fair and effective emotion prediction in understanding human behavior through text.

**Supplementary Information:**

The online version contains supplementary material available at 10.1038/s41598-026-46749-7.

## Introduction

### Reducing social biases in text-based emotion prediction using semantic blinding and semantic propagation graph neural networks

The automated assessment of emotional content in textual data, or emotion prediction (EP) has revolutionized research across the social sciences, enabling applications such as suicide risk prediction from text messages^1^, analysis of historical well-being trends^2^, political election forecasting^3^,and monitoring global emotional responses during crises like the COVID-19 pandemic^4^. Thanks to EP researchers gained access to an extensive array of authentic data on human emotions, as vast as the multitude of texts available on the internet.

However, current methods for emotion assessment have notable limitations. Transformer-based architectures, and other machine learning models — while being recommended for their high performance^5^, are at the same time susceptible to inheriting social biases from their training data, including gender, racial, ageist, and political biases^6–8^. For instance, Kiritchenko and Mohammad^6^surveyed 219 automatic emotion prediction systems 75% of which showed signs of significant racial and/or gender bias. A more targeted investigation conducted found that a model trained on annotated political texts exhibited biases aligned with the political orientation of the annotators. Removing bias relevant items from the training data reduced these biases, implying that the annotations were their source of origin^8^. As these findings highlight, addressing bias in emotion modeling has become an essential challenge for emotion prediction research.

The solutions to the problem of bias available so far have significant drawbacks. One of them – the balancing of the annotator group in terms of bias, is imperfect as, aside from the labor required to find the right people, there always exists a risk of the existence of a bias that was not accounted for. The proposed alternative so far has been the use of simpler models, such as those based on lexicons (also called norms or dictionaries), which are long lists of manually selected words annotated for their emotional information^9^. The most basic lexicon approaches rely on simply looking up the emotional value of each available word in a text and averaging the results. Unfortunately, this approach works well only for very simple texts, as it does not consider syntactic information. An example here is negation, which can transform the meaning of a word in a sentence but goes unnoticed by simple dictionaries.

More complex alternatives rely on hard coded rules to handle syntactic dependencies. Examples of such approaches are the VADER (Valence Aware Dictionary and Sentiment Reasoner), and Emotlas^10,11^. Both techniques rely on dictionaries combined with hard-coded rules that were arrived at through examination of sentence structures. Rules such as “if the negation is three words away from an emotionally loaded term, flip the emotional loading of the term” allow those models to handle negations and other semantic structures beyond the reach of normal lexicons. Their performance however rarely approaches that of pretrained transformers and the degree of generalization to languages different than English is questionable, due to different syntactic patterns being present in languages further away on the language phylogenetic tree.

Even more advanced solutions that try to debias transformer-based emotion models by targeted debiasing, such as creating counterfactual training examples^12^, adversarial^13^, post-hoc removal of features related to specific guarded attributes^14^, or plain removal of training data instances that might affect model fairness^8^, all require the user to specify which specific dimension of bias, or fairness they are interested in safeguarding. Therefore, they are susceptible to the same problem as annotator group balancing – difficulty in specifying all relevant dimensions of bias.

### The proposed solution

To provide a more robust and general solution, this paper presents the Semantic Propagation Graph Neural Network (SProp GNN), a supervised approach that bridges the methodological gap between simple lexicon-based methods and complex black-box models providing high performance that is robust to training data bias. This approach uses syntactic relationships within sentences to create graphs enhanced with emotion information at the word level. The SProp GNN is then trained on these graphs, providing emotion predictions at text level. The risk of bias propagation is reduced by purposefully blinding the model to semantic information that it could otherwise overfit.

**Fig. 1:**
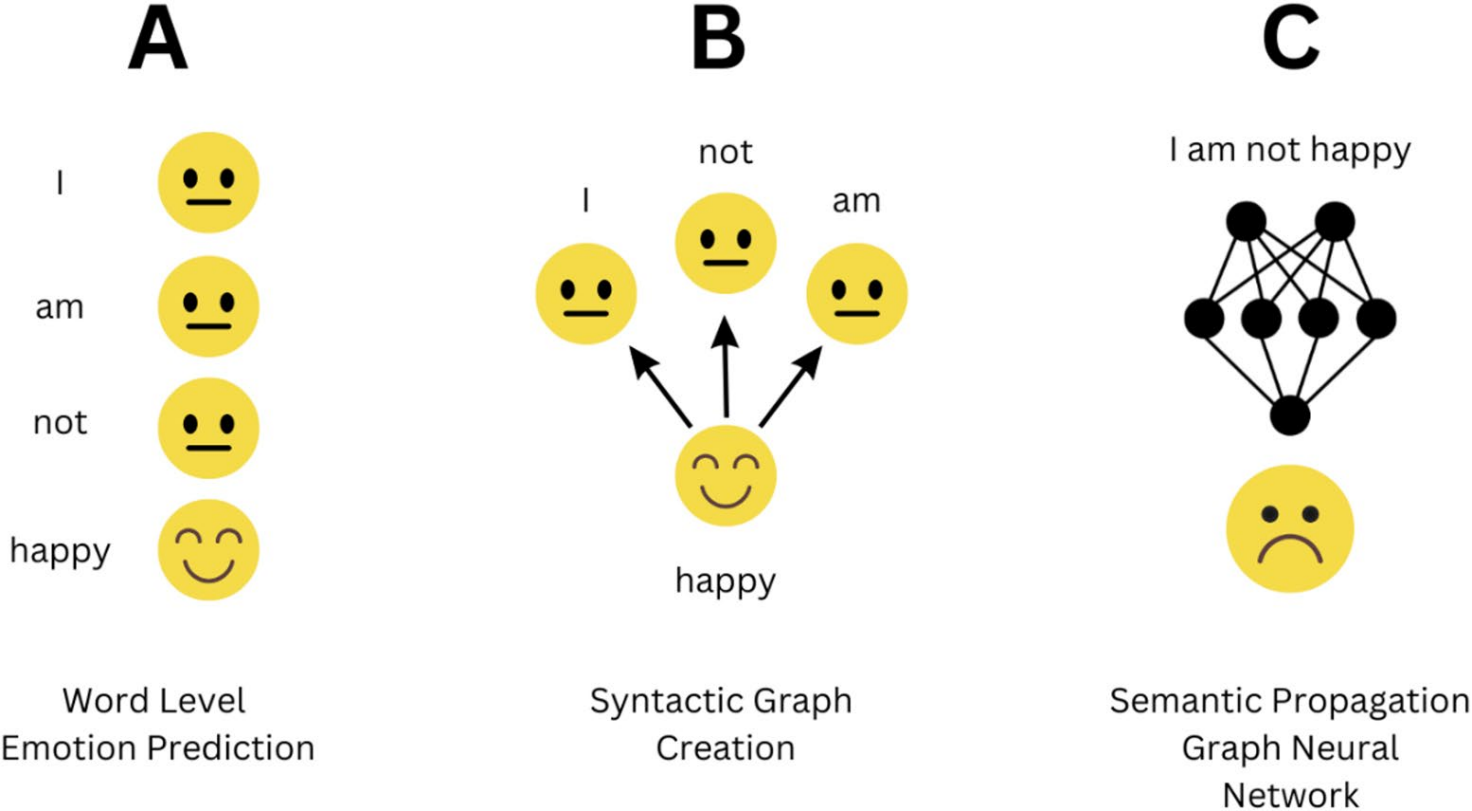
Steps of the emotion prediction pipeline.

The SProp GNN emotion prediction pipeline can be split into three distinct stages (as seen on Fig. 1.):

Stage A – word level emotion prediction - The emotional value of each word is identified.

Stage B – syntactic graph creation – The sentence is transformed into a graph that reflects the syntactic connections between words.

Stage C – semantic propagation graph neural network – A specialized neural network processes these graphs, along with the emotional information of singular words, to predict the overall emotional meaning of a text, without relying on the direct knowledge of the words that constitute it.

Part A portrays words from a sentence “I am not happy” split into word-level tokens and associated with their respective emotional loads symbolized with emojis; part B shows a syntactic graph created based on the syntactic structure of the sentence; Part C shows the initial sentence being processed using the SProp GNN and resulting in a final negative sentiment score. The proposed approach of selectively withholding specific semantic information from the model can be termed *Semantic Blinding*. This term was chosen because the method deliberately limits (or blinds) the model’s access to semantic details to prevent it from associating emotional predictions with specific words or concepts that could introduce unwanted biases. By focusing exclusively on syntactic relationships and word-level emotional cues, semantic blinding ensures that the model’s emotional assessments are free from training data biases related to specific groups or subjects. This technique, therefore, enhances the model’s capacity to generalize across varied text sources without inheriting unintended, potentially harmful associations, providing a robust and unbiased tool for emotion prediction. Furthermore, given that it does not require the user to specify which dimensions of fairness they are concerned with; it bypasses the problems of earlier methods.

### Brief introduction to graph neural networks

Graphs are mathematical structures used to represent entities (nodes) and their relationships (edges), providing a powerful framework for analyzing structured data^15^. In the context of natural language processing (NLP), a sentence can be represented as a graph where words serve as nodes, and edges capture syntactic or semantic relationships, such as dependencies between words. Graph Neural Networks (GNNs) extend this framework by applying deep learning techniques to graphs, enabling models to learn from the relationships and structures inherent in the data. Unlike traditional neural networks, which operate on fixed-size inputs like vectors or grids, GNNs analyze the connectivity patterns and features of nodes and edges to perform tasks such as classification, prediction, or clustering^15^. For example, GNNs have been successfully used in applications ranging from molecule property prediction in chemistry to fraud detection in financial networks^16,17^. They have also gained recognition in emotion prediction applications (for a comprehensive review^18^, however since the main aim of the previous methods was to maximize the predictive performance of their approaches, none of them limited the amount of information that the model received, as is done in the case of Semantic Blinding.

### The contents of the paper

Through comparative experiments, this paper demonstrates that the SProp GNN outperforms traditional lexicon-based models as well as lexicon-based alternatives across both discrete and dimensional emotion prediction tasks in English and Polish. It closely approaches transformer-based model accuracy in both languages, and task types offering a compelling alternative to biased black-box models. Furthermore, the paper provides detailed statistical and theoretical evidence that the SProp GNN is robust to the biases shown in previous research.

## Results

### The GoEmotions dataset

In the task of discrete emotion prediction conducted on the GoEmotions dataset^19^, the Semantic Propagation GNN generally outperforms the Emotlas approach across the three key performance metrics: accuracy, precision, and recall (See Table 1.). Here, accuracy measures how often the model’s predictions are correct overall, precision assesses the proportion of correct positive predictions among all positive predictions made, and recall evaluates the model’s ability to identify all actual instances of each emotion. The only exceptions are in the accuracy metric for the emotion of sadness, where Emo Atlas slightly exceeds the GNN.

While the SProp GNN shows better performance than Emo Atlas, both methods are generally surpassed by their transformer-based counterpart. The RoBERTa model, which leverages advanced language representations, outperforms both the Emo Atlas and the Semantic Propagation GNN across all emotions and metrics. However, the GNN is not far behind RoBERTa , achieving a mean accuracy difference of only 5.70% points, compared to a difference of 10.35% points between RoBERTa and the Emo Atlas.

Similarly, for precision, the average difference between RoBERTa and the GNN is 17.05% points, whereas the difference between RoBERTa and the Emo Atlas is 26.77% points. In terms of recall, the average difference between RoBERTa and the GNN is 20.81% points, while the difference between RoBERTa and the Emo Atlas is 45.12% points. These results indicate that while the GNN approaches the performance of RoBERTa , the Emotlas method lags significantly behind in all three metrics.

**Table 1:** Performance results on the GoEmotion dataset.

Emotion	Accuracy score %			Precision score %			Recall score %		
	RoBERTa	Emo	SProp	RoBERTa	Emo	SProp	RoBERTa	Emo	SProp
Anger	91.3	0.68	**80.0**	86.7	0.51	**65.9**	87.8	0.45	**82.3**
Disgust	93.9	0.87	**88.9**	78.2	0.44	**50.7**	63.0	0.25	**35.1**
Fear	94.7	0.91	**93.2**	71.2	0.61	**72.1**	85.6	0.41	**59.6**
Joy	97.3	0.81	**92.2**	93.0	0.56	**76.4**	90.7	0.20	**77.8**
Sadness	94.8	**0.90**	88.9	81.4	0.59	**61.5**	80.2	0.55	**48.9**
Surprise	96.1	0.89	**90.7**	85.1	0.64	**66.7**	86.4	0.37	**65.1**

The emotion categories had to be limited to those presented in the table both due to lexicon availability and Emotlas emotion coverage. The results written in bold pinpoint the best performance in a given metric out of the two alternatives to transformers. Model codes: RoBERTa – finetuned transformer; Emo – the Emo Atlas approach; SProp – Semantic Propagation GNN model.

### The EmoBank dataset

The task of sentence level emotion prediction was run on the EmoBank dataset^20^, on which the SProp GNN outperformed both the lexicon approach and the Vader approach (see Table 2.). While RoBERTa achieved higher scores than the SProp GNN, the degree of difference varied between predicted metrics. While in the case of valence the difference amounted to 0.13 points, for arousal it was as low as 0.02.

**Table 2:** Performance results on the emobank dataset (Pearson’s Correlation).

Metric	RoBERTa	Lexicon	Vader	SProp
Valence	0.75	0.45	0.46	**0.62**
Arousal	0.48	0.25	-	**0.45**

The results written in bold pinpoint the best performance, measured using the Pearson’s correlation, in a given metric out of the three alternatives to transformers. Model codes: RoBERTa – finetuned transformer; vader – the Vader approach; lexicon – the lexicon approach; SProp – Semantic Propagation GNN model. The lexicon score has been calculated after pruning stopwords.

### The Polish political dataset

### Political bias results

Finally, the Polish political dataset^21^ was used to test the model performance on a mixed set of both multiple and single sentence texts. Here, the SProp outperformed its lexicon counterpart yet again (See Table 3.). Unsurprisingly, it was at the same time worse at predicting emotion scores than the RoBERTa model by 0.16 points in the case of valence, and 0.13 points in the case of arousal.

**Table 3:** Performance results on the polish political dataset (Pearson’s Correlation).

Metric	RoBERTa	Lexicon	SProp
Valence	0.88	0.57	**0.72**
Arousal	0.75	0.33	**0.62**

The results written in bold pinpoint the best performance in a given metric, measured using the Pearson’s correlation, out of the two alternatives to transformers. Model codes: RoBERTa – finetuned transformer; lexicon – the lexicon approach; SProp – Semantic Propagation GNN model. The lexicon score has been calculated after pruning stopwords.

### Political bias results

#### Approach 2: assessing bias reduction in the SProp model

The beta coefficient for bias was *β* = −0.78, with a permutation p-value significant at *p* = 0.012. This result suggests that the SProp model’s valence predictions exhibit a substantially lower level of bias compared to those of the transformer model. The negative value of the coefficient indicates an inverse relationship, suggesting that the bias introduced by political affiliation and gender in the original model has been largely mitigated in the SProp GNN model. Given the potential for real-world measurement variability and the effects of regression dilution, a coefficient of −0.78 strongly implies that the SProp GNN has no significant residual bias from the original model, or that any remaining bias is minor and unlikely to have practical significance.

Table 4 presents the regression results, including the adjusted R² of 0.535, indicating a moderate fit for the model. While the dummy variables for sentence types were included to account for systematic differences between neutral and political sentences, their specific coefficients are not central to the interpretation of bias reduction. The key finding remains that the SProp GNN model shows a marked reduction in bias, as evidenced by the negative and significant beta coefficient for the bias factor.

**Table 4:** Regression models – differences in valence.

	Transformer			Semantic propagation GNN		
	Dependent variable: valence of:					
	Names only (1)	Neutral sentences (2)	Political Sentences (3)	Names only (1)	Neutral Sentences (2)	Political Sentences (3)
intercept	45.40*** (0.67)	48.61*** (0.92)	43.89*** (0.26)	53.59*** (1.87)	55.02*** (0.71)	44.96*** (0.49)
3D	5.73** (2.37)	9.09** (3.26)	2.72** (0.93)	2.33 (6.61)	1.44 (2.52)	0.73 (1.71)
K	6.15* (3.10)	8.37* (4.28)	2.56* (1.22)	3.85 (8.67)	0.83 (3.30)	1.02 (2.24)
KO	5.83*** (1.31)	3.71* (1.80)	2.30** (0.51)	0.93 (3.66)	0.18 (1.39)	0.60 (0.94)
Left	3.03 (2.56)	5.46 (3.53)	2.51** (1.01)	−3.75 (7.17)	−3.02 (2.73)	−1.14 (1.85)
gender	9.77** (3.48)	10.10* (4.80)	1.88 (1.37)	8.10 (9.74)	3.19 (3.71)	2.11 (2.52)
Observations	22	22	22	22	22	22
R²	0.665	0.520	0.662	0.077	0.135	0.103
Adjusted R²	0.547	0.370	0.556	−0.211	−0.135	−0.178
Residual std. error	3.38 (df = 16)	5.21 (df = 16)	1.29 (df = 16)	6.69 (df = 16)	2.75 (df = 16)	1.70 (df = 16)
P-value (permutation)	0.008***	0.049**	0.018**	0.885	0.686	0.793

*p < 0.1; **p < 0.05; ***p < 0.01 (*t-*test)

Zjednoczona Prawica (ruling party) as intercept, gender: woman = 1, man = 0

*Abbreviations*: K – Konfederacja, 3D – Trzecia Droga, KO – Koalicja Obywatelska, Left – Nowa Lewica

#### Approach 3: comparing differences between models

The beta coefficient in this analysis explained a significant portion of the variance in valence prediction differences between the two models, with β = 0.78 and a permutation p-value of *p* = 0.028. This finding strongly supports the conclusion that the SProp model propagates substantially less bias related to political affiliation and gender compared to the transformer model. The positive and significant coefficient indicates that the difference in predictions between the two models is systematically related to the bias factors identified in the transformer model, further highlighting that the SProp GNN effectively reduces the bias originally observed.

Table 5 presents the results of this regression. The intercept (−8.18, *p* < 0.001) represents the baseline difference between the models’ predictions, while the bias coefficient (*β* = 0.78, *p* < 0.01) accounts for a significant portion of the variance. The inclusion of dummy variables for neutral and political sentences adjusts for systematic differences across stimulus types. While these coefficients (neutral: *β* = 2.14, *p* < 0.1; political: *β* = 7.11, *p* < 0.001) suggest some variation in the magnitude of prediction differences based on sentence type, the primary finding lies in the bias coefficient itself, which demonstrates the central role of bias reduction in distinguishing the SProp GNN’s predictions from those of the transformer model.

**Table 5:** Regression model - testing for reduction in bias.

Dependent variable: Difference between Transformer and SProp GNN Valence Predictions	
intercept	−8.18***(0.94)
Bias	0.78*** (0.19)
Neutral sentences	2.14* (1.19)
Political entences	7.11*** (1.21)
Observations	66
R^2^	0.415
Adjusted R²	0.386
Residual std. error	4.368 (df = 62)

*p < 0.1; **p < 0.05; ***p < 0.01 (*t-*test).

The analysis tested three hypotheses to evaluate the bias robustness of the SProp GNN model compared to the transformer model. The null hypothesis about the SProp GNN being robust to bias was retained, but the results were inconclusive. The second hypothesis, which posited that the SProp model’s bias is equivalent to the transformer model’s, was rejected, showing a significant reduction in bias. The third hypothesis, testing whether prediction differences between the models are related to bias factors, was also rejected, confirming that the SProp model mitigates the biases observed in the transformer model. These results strongly support the conclusion that the SProp GNN substantially reduces bias and is a reliable alternative for unbiased emotion prediction.

### Explainability

**Fig. 2:**
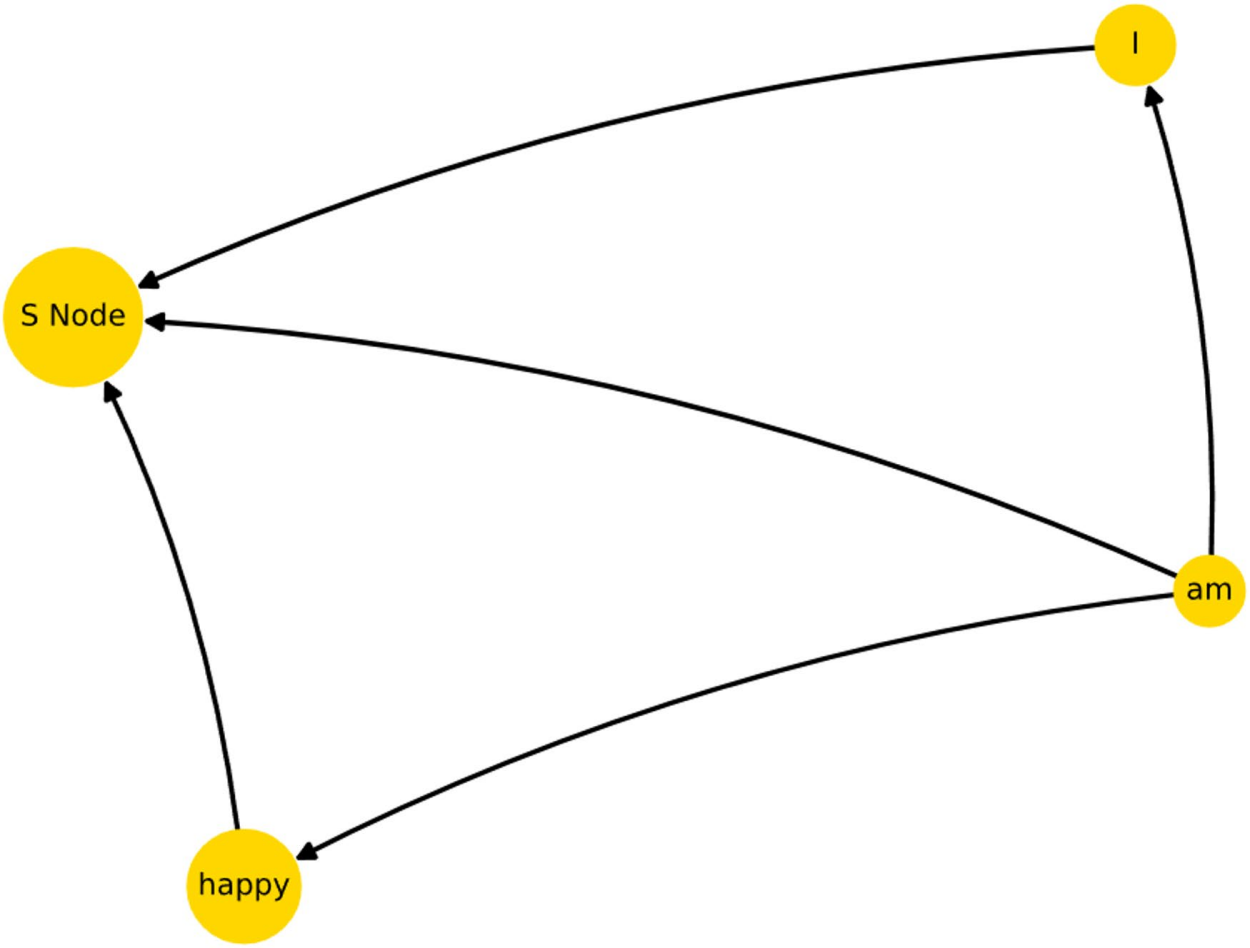
The explanatory graph for sentence “I am happy”.

**Fig. 3:**
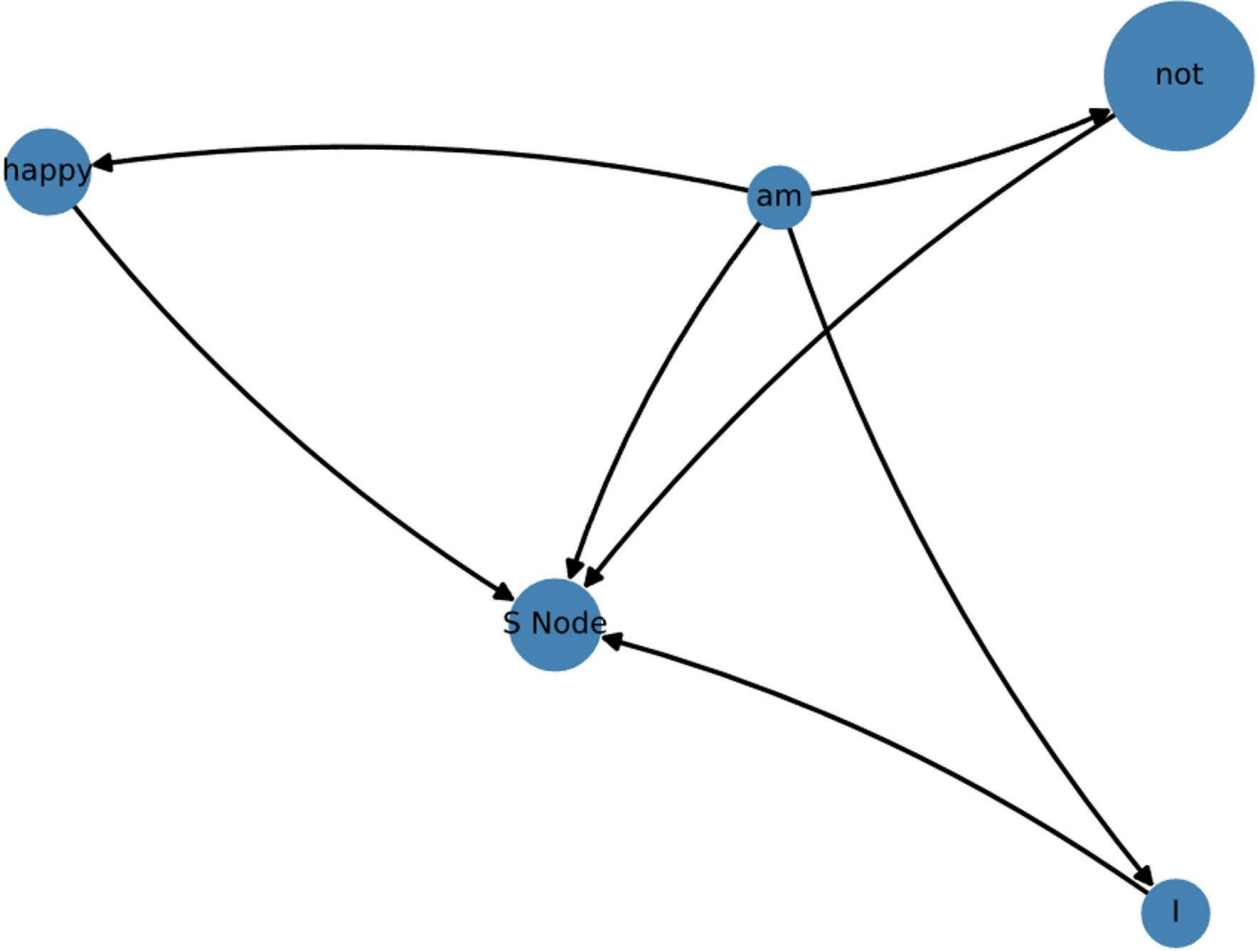
The explanatory graph for sentence “I am not happy”.

The SProp model trained on the EmoBank dataset was used to assess the valence of two sentences: “I am happy”, and “I am not happy”. The former sentence received a valence prediction of 0.68, and the latter, a valence prediction of 0.43 indicating that the model can take negation into account and appropriately modify its prediction. Figures 2, and 3 depict an abstract representation of what happened inside the model during the prediction. Each figure visualizes the dependency graph of an input sentence as constructed by the spaCy parser, where nodes correspond to tokens and directed edges correspond to syntactic dependency relations used for semantic propagation by the SProp GNN. The size of each node is proportional to the attention weight assigned to that node by the model, computed from the attention mechanism and rescaled for visualization purposes.

The size of the nodes represents the degree to which the model attended to a given node’s feature when extracting information from the graph. The S Node refers to the sentence node. In the case of the first sentence (Fig. 2), the model relied on the emotional information from the sentence node, and the node that contained the emotional features of the word “happy”. This is expected as the sentence node contains information passed from all of the word nodes in the sentence, while the “happy” node is an adjective, and so often conveys emotional information. In the case of the second sentence, however (Fig. 3) the model paid significantly more attention to the “not” node features, indicating that it learned that negation could reverse the emotional load of a sentence.

However, as can be seen in Fig. 3, the pathway of emotional propagation from the word “happy” does not include the negation node. This means that the mechanism through which SProp GNN operates is partially based on heuristics, rather than just on the propagation of emotional information through the syntactic graph. To test this, a longer sentence including a negation that should not modulate the emotional information was processed by the model. The sentence “I am happy, and not tall” was given a valence prediction of 0.431, while the same sentence “I am happy, and tall” received a rating of 0.69.

The scaling factors used to propagate emotional information through the syntactic graph were not visualized, as they do not convey a lot of information. This is due to their high dimensionality. In the current models, the dimension of the scaling factors is equal to the hidden dimension of the SProp Layer – 512. This means that their average likely obscures a lot of information about their underlying mechanisms.

#### Approach 1: replicating the original regression procedure

The replication of regressions performed in the original bias study^8^ yielded no significant results for the SProp GNN model (see Table 6). Neither political affiliation nor gender explained any meaningful variance in the model’s valence predictions across any of the stimuli categories: names, neutral sentences, and political sentences. This outcome contrasts with the results obtained for the transformer model in the original study, where political affiliation and gender were significant predictors, explaining 66% of the variance in valence for political sentences, 52% for neutral sentences, and 67% for names alone. For the SProp model, these same predictors explained a negligible proportion of the variance, as shown by the R² values of 0.077, 0.135, and 0.103, which are accompanied by non-significant permutation test p-values.

In Table 6, the results are presented for both models. The regression intercepts represent the predicted valence for the reference group, which is Zjednoczona Prawica (the ruling party) and male politicians, while the coefficients for political affiliation indicate how much valence changes for other groups (e.g., Konfederacja, Koalicja Obywatelska, etc.). For the transformer model, the political affiliation coefficients are consistently significant across all stimulus types, confirming substantial bias in its predictions. For example, the valence associated with Koalicja Obywatelska is consistently higher than that for Zjednoczona Prawica, with coefficients such as 5.83 (neutral sentences) and 2.30 (names only), both significant at *p* < 0.05. Gender also has a notable influence in the transformer model, with a coefficient of 9.77 for neutral sentences, indicating that valence predictions for women are substantially more positive than for men in this category.

In contrast, the SProp GNN model shows no significant coefficients for political affiliation or gender in any stimulus category. For instance, the coefficient for Koalicja Obywatelska is close to zero (e.g., 0.18 for neutral sentences, 0.93 for names, and 0.60 for political sentences) and accompanied by p-values well above 0.05. Similarly, the gender coefficient is 3.19 for neutral sentences, 2.11 for political sentences, and while as high as 8.10 for names only, it is not statistically significant. These results suggest that the SProp GNN model’s predictions are less systematically influenced by political affiliation and gender, highlighting a potential reduction in bias compared to the transformer model.

Despite these findings, it is important to interpret the results with caution. While the lack of statistical significance in the SProp GNN model suggests an absence of systematic bias, this alone does not confirm that the model is entirely unbiased. The low explanatory power of the regressions and the non-significant results may also reflect limitations in the sensitivity of the statistical tests or the sample size, rather than the true absence of bias. Moreover, differences in residual variance and standard errors between the models indicate that additional factors may be influencing the outcomes. Specifically, despite the SProp GNN model showing no significant results, some of the beta-coefficients were similar to those coming from the original model (e.g. 9.77 for gender in the original, and 8.10 in the SProp GNN case), and thus given the higher standard deviations (e.g. 3.48 for gender in the original versus 9.74 for the SProp GNN), this result could be a type 2 error. At the same time, however the stability of the coefficients for other sources of bias wasn’t as high (e.g. coefficient of 3.03 vs. −3.75 for the Left dummy) and given that the different types of SProp GNN models explained close to 0% of variance these patterns are most likely completely spurious. Therefore, complementary analyses, such as those presented in later sections, are essential to provide a more comprehensive understanding of bias reduction in the SProp GNN model.

**Table 6:** Regression model – explaining the difference in predictions.

Dependent variable: Bias Reduced SProp GNN Valence Predictions	
Intercept	53.58***(0.96)
Bias	−0.78*** (0.20)
Neutral sentences	−1.07 (1.21)
Political sentences	−8.62*** (1.24)
Observations	66
R^2^	0.557
Adjusted R²	0.535
Residual std. error	4.04 (df = 62)

Note. *p < 0.1; **p < 0.05; ***p < 0.01 (*t-*test).

## Discussion

The present study introduces the Semantic Propagation Graph Neural Network (SProp GNN) as a novel approach to emotion prediction, addressing the critical issue of bias propagation inherent in machine learning based models. The SProp GNN significantly outperformed other lexicon-based alternatives across two different languages (English and Polish) and two distinct emotion prediction tasks, namely discrete and dimensional emotion prediction. It’s ability to utilize the syntactic structure of sentences embedded with emotional information on the single word level allowed it to bridge the gap between simple lexicon-based methods and complex black-box models.

The main contribution of this work is the demonstrable reduction of bias in emotion prediction. Such bias can lead to unfair or discriminatory outcomes, both in real world applications such as mental health assessments^22^, hiring processes^23^, or criminal justice systems^24^, as well as in the academia, where scientific conclusions are required to be fair and objective. The statistical evidence presented shows that the SProp GNN propagates at least significantly less bias than its transformer-based counterpart. This evidence, coupled with the sole fact that the SProp GNN simply does not have access to any bias information it could overfit, since it only processes the syntactic structure of the sentence coupled with external emotional ratings and not words directly, warrants a claim that it is robust to training data bias. By effectively mitigating bias, the SProp GNN not only enhances the fairness and ethical standing of emotion prediction tools but also increases their reliability across diverse populations. This is particularly crucial in a global context where texts may reflect a wide array of cultural, social, and individual differences. The ability of the SProp GNN to provide more objective emotional assessments can contribute to more equitable decision-making processes in applications that rely on emotion prediction.

While the SProp GNN performs slightly worse than pretrained transformers, it constitutes a viable alternative in applications where objective emotional assessment is of key importance. The development of the SProp GNN highlights the inherent trade-offs between model interpretability, performance, and bias mitigation. Transformer-based models often achieve higher accuracy due to their ability to capture complex semantic nuances; however, they also tend to act as black boxes, making it difficult to understand or control the sources of their predictions, including biases. In contrast, the SProp GNN offers a more interpretable architecture, allowing for greater transparency in how predictions are made. Although there is a slight decrease in performance compared to transformers - a mean accuracy difference of 5.70% points on the GoEmotions dataset, for instance - this trade-off may be acceptable or even preferable in contexts where interpretability and bias reduction are prioritized over marginal gains in accuracy. Researchers should therefore consider whether in the context of their specific application the small accuracy drop outweighs potential fairness concerns.

The bias robustness of the SProp GNN makes it particularly suitable for applications where fairness and objectivity are paramount. For instance, in the analysis of social media data for public health monitoring^25^, using a model that minimizes bias ensures that interventions are based on accurate representations of population emotions without skewing toward or against specific groups. In mental health contexts, such as suicide risk prediction from text messages^1^, unbiased emotion prediction can lead to more accurate assessments and timely interventions. Increasingly popular tools that analyze student feedback^26^ can also benefit from unbiased emotion assessments to foster an inclusive learning environment. In all these cases and more, the SProp GNN’s ability to deliver high-performance emotion predictions while mitigating bias is of significant practical value.

The SProp GNN seems susceptible to simplistic heuristics, as shown in the explainability section, where the model did not fully capture the nuanced role of negations in complex sentences. This potential discrepancy between the good performance results of the model and its inability to pick up on syntactic cues that are easily understandable to a human can be explained by the low frequency of such sentence structures in the tested datasets and their overall rarity. The datasets used are diverse in language and task types, but they may not encompass the full spectrum of linguistic structures and expressions found in real-world texts. This could limit the generalizability of the SProp GNN to other languages or dialects not represented in the training data. Therefore, researchers should consider the linguistic complexity of the data they want to analyze using SProp GNN before committing to this method. Furthermore, an important limitation of the present comparison is the absence of transformer models debiased with respect to explicitly defined protected attributes^12–14^. While such models represent a powerful complementary approach, they address a different problem formulation than the bias-agnostic strategy proposed here.

Future studies can further improve the architecture of the SProp GNN, shrinking the gap between its performance and that of other potentially biased models. One potential avenue for further exploration could be a modification of the syntactic graph creation algorithm. While the syntactic pathways extracted using the spaCy package^27^ provide useful information about the structure of sentences, a more tailored model that would map the pathway of emotional information propagation directly could achieve even better results, potentially reducing the model’s reliance on heuristics. Expanding the training datasets to include more syntactically diverse sentences could help the model learn to handle complex linguistic structures more effectively.

When it comes to the applicability of the SProp GNN to other languages, specifically those that are more syntactically distant from the two western languages used to benchmark the method, it should be directly dependent on the availability of syntactic parsers and norm-extrapolation models. The spaCy package already provides parsers for many non-indo European languages such as Japanese, Chinese and others. On the other hand, given the considerable availability of semantic norms, and a straightforward method of training transformer-based norm-extrapolators^28^, the word level emotional prediction should not be an issue either. These claims are, however, hypothetical and will have to be tested in future studies.

Furthermore, the model’s reliance on lexicons, or norm-extrapolation models could introduce bias present on the word level. Despite the lack of context when annotating emotions at word level, there is still a small possibility that the annotators for the lexicons impacted some sorts of biases on the emotion dictionary. This type of bias, however, can usually be easily explored using the lexicon in question, and its mitigation is as simple as equalizing the emotional load of words that convey it. In circumstances where a specific type of bias could directly impact the conclusions of a study, checking the lexicon for its presence before the use of the SProp GNN is advisable.

The proposed approach of selectively withholding specific semantic information from the model, termed semantic blinding, is a technique that deliberately limits the model’s access to semantic details. By preventing the model from associating emotional predictions with specific words or concepts that could introduce unwanted biases, semantic blinding ensures that the model’s emotional assessments are free from training data biases related to specific groups or subjects. This technique presents exciting opportunities for future research. It could be extended to other natural language processing tasks where bias could be a concern, such as text-classification. Exploring how semantic blinding can be integrated with transformer-based architecture might also yield models that combine the high performance of transformers with the bias mitigation benefits of the SProp GNN. Additionally, further investigation into the types of semantic information that can be withheld without significantly impacting performance could lead to the development of more robust and fair NLP models across various domains.

From a practical standpoint, deploying the SProp GNN in real-world applications offers significant advantages in terms of computational efficiency and scalability due to its substantially smaller model size when compared to its transformer-based counterparts. Specifically, the SProp GNN trained on the EmoBank dataset consists of approximately 1.5 million parameters, while the transformer model trained for the same task comprises about 125 million parameters. This significant reduction in model size - over 20 times smaller - translates to lower computational overhead and faster processing times, making the SProp GNN more suitable for deployment on devices with limited resources or for applications requiring real-time analysis. For such applications, the word level prediction stage of the model could be done prior to inference time by generating a very large emotional dictionary a priori. From an academic standpoint, this translates to accessibility for researchers without access to high-performance computing resources. The reduced memory and processing requirements mean that the SProp GNN can be trained and deployed on standard hardware, broadening the scope of researchers who can experiment with and apply this model. This adaptability and efficiency make the SProp GNN a practical and accessible alternative to transformer-based models in emotion prediction tasks.

To allow other researchers to replicate the analyses presented in the current paper and use the SProp GNN architecture for their research, the code, along with detailed comments for this paper has been made available at a GitHub repository (https://github.com/hplisiecki/Semantic-Propagation-GNN). Additionally, the Technical Appendix should serve as additional guide for those willing to apply and further develop the methods here presented.

In conclusion, SProp GNN represents a significant step forward in developing emotion prediction models that prioritize fairness and interpretability without substantial sacrifices in performance. The evidence demonstrates that the SProp GNN not only approaches the accuracy of transformer-based models but also at least significantly reduces the propagation of biases. This coupled with the fact that the model does not possess the ability to overfit specific words points towards near total bias reduction. The method can therefore be applied to any emotion prediction task with the benefit of reducing social bias, however lexical complexity of the analyzed text, and norm extrapolation model availability should be considered when using it. While there is room for improvement, particularly in handling complex syntactic structures and expanding language coverage, the SProp GNN lays the groundwork for future advancements in unbiased and interpretable emotion prediction. Future work focused on enhancing the model’s architecture, expanding its applicability, and refining the semantic blinding technique holds the promise of further bridging the gap between high performance and bias mitigation in natural language processing.

## Methods

### The emotion prediction system

The method proposed by the current paper essentially combines the use of three different machine learning based approaches, which process the text sequentially.

#### Word level emotion prediction

The task of the first model is to predict the emotional value of the words in the text. This task, also seen as norm-, or lexicon-extrapolation is currently best attempted using transformer-based models^28^. For the purposes of the current paper, either existing pretrained transformers norm extrapolation models are used, or new ones are trained when no off-the-shelf solutions are available (for more information see the Technical Appendix; there Supplementary Tables 1 and 2 report the norm extrapolation model performances). This stage results in emotion estimates for each separate word in each text.

#### Creation of the syntactic graph

The text is then divided into sentences, and these sentences are analyzed using the *spaCy*package^27^. This software uses machine learning algorithms and linguistic rules to parse text, creating detailed syntactic structures for each sentence. *SpaCy* generates dependency graphs, which represent the relationships between words, as well as dependency labels (e.g. negations) and part-of-speech (POS) tags (e.g. verb). If a text consists of multiple sentences, these are linked back together using dedicated sentence nodes. This procedure allows the framework to capture the structure of each text, providing a type of scaffolding for the SProp GNN to propagate word-level emotional information through. This stage results in the creation of a syntactic graph, enriched with information about the part-of-speech categories, and emotions from the first stage, at the node level, and dependency labels at the edge level.

#### Semantic propagation graph neural network

The final part of the pipeline is the SProp GNN, a neural network model designed to propagate emotional information extracted in the first stage of the pipeline, through the graph generated in the second stage. SProp GNN can be split into three main components: a custom SProp (GNN) layer, an attention pooling mechanism, and linear output layers.

##### Custom graph neural network layer

 At the heart of the model is the custom Semantic Propagation Layer. This layer operates on the dependency graphs generated by spaCy^27^, where each node represents a word with associated features, and edges represent syntactic relationships between words. The Semantic Propagation Layer integrates information from the word node features (earlier predicted emotional load, and part-of-speech tags) and edge features (such as dependency types) to compute a scaling factor for each of its edges. It then propagates the emotional information from word nodes along those edges, scaling them accordingly. The hope here is that by doing so, it can model the propagation of emotional information through the sentence.

##### Attention pooling mechanism

 After the graph has been processed by the SProp GNN layer, the model employs an attention-based pooling mechanism^29^. This component aggregates the information from all nodes in the graph to create a single, fixed-size vector representation of the entire text. The attention mechanism assigns different weights to word nodes based on their relevance, effectively allowing the model to focus on the most significant words and relationships when forming this overall representation.

##### Linear output layers

 The aggregated text representation is then passed through multiple linear layers. These layers transform the high-dimensional embedding into a scalar value between 0 and 1, corresponding to the predicted score for each predicted emotional metric. By having separate output layers for each metric, the model can simultaneously make multiple predictions, each tailored to the unique aspects of the respective psychological construct. This is different for discrete classification, where the layers transform the embedding to a vector of size equal to the number of predicted classes. This vector, when transformed, becomes an array of class probabilities.

The SProp GNN model processes text by first constructing a rich representation of its syntactic and semantic structure using the SProp layer. It then distills this information into a concise and meaningful summary via attention pooling. Finally, it translates this summary into actionable predictions through the linear output layers. Before prediction, this model has to be trained on a dataset of texts, with annotated emotional metrics in the form of either emotion intensities, or discrete emotion classes. As the model does not have direct access to the words with regards to which people exhibit social bias (e.g. certain politicians, gender information etc.), it cannot learn the association between them and the biased emotion estimates. Therefore, the biased part of an emotion estimate is from its perspective indistinguishable from noise, as it has no systematic relationship with the input data. This renders the model blind to the socially sensitive features of the input, therefore rendering it agnostic with regards to social biases. For a more detailed description of the model architecture, as well as complete pipeline pseudocode see the Technical Appendix.

### Comparative experiments

The SProp model has been tested on three separate datasets, the GoEmotions dataset^19^, the EmoBank dataset^20^,and the dataset used in the Plisiecki and colleagues political bias study^8,21^. These datasets cover two languages (Polish and English), and two different emotion prediction tasks (categorical, and continuous emotion prediction).

####  The GoEmotions dataset

The GoEmotions dataset, developed by Google researchers^19^, consists of around 58,000 English Reddit comments annotated with 28 distinct emotions, totaling over 210,000 annotations. Sourced from a Reddit data dump spanning 2005 to early 2019, the dataset includes comments from diverse subreddits, balanced by capping comment counts from the most popular communities and sampling evenly across others. Emotion categories were curated based on psychological research to represent a broad but non-overlapping range of emotions. Each comment received annotations from three English-speaking raters from India, with additional raters assigned when agreement was low.

#### The EmoBank dataset

The EmoBank dataset^20^, consists of 10,062 English sentences from sources like news, blogs, fiction, and letters, annotated along three emotional dimensions: Valence, Arousal, and Dominance (VAD). Each sentence was rated by five annotators from the crowdsourcing platform CrowdFlower for both *writer* and *reader*perspectives on a 5-point scale, giving insights into both expressed and perceived emotions. In accordance with the recommendations of the researchers, the current paper uses the version of the dataset with the weighted average of the reader and writer perspective labels provided at their online repository^30^..

#### The polish political dataset

The Polish Political dataset^21^ includes 1.25 million Polish social media posts from journalists, politicians, NGOs, and general users. The emotionally neutral texts were filtered out using lexical norms on valence, arousal, and dominance. The final 10,000 texts were annotated by 20 psychology-trained annotators on six emotions (happiness, sadness, anger, disgust, fear, and pride) and two dimensions (valence and arousal) using a 5-point scale. Each annotator completed five weekly sets of 100 randomly assigned texts, ensuring each text was labeled by five raters for reliable coverage and minimizing cognitive fatigue over the five-week process. The resulting scores were averaged to create an intensity score for each text – emotion pair.

#### Dataset preparation

Each of the datasets was first prepared by either calculating the most voted emotion category in the case of GoEmotions or normalizing the intensity of annotations to 0 to 1 range in the case of the two continuous datasets. Each dataset was then split into the training, evaluation, and test subsets in a proportion of 8:1:1, except for the Polish dataset, for which the split dataset was taken from the original paper^21^. For more information about the preparation of the datasets and the datasets themselves see the Technical Appendix.

### Comparative approaches

The aforementioned datasets are used to compare the SProp model’s performance to four alternative methods. The first three methods rely on lexicons, and as such are resilient to annotator bias. For the proposed framework to become a preferred alternative to them, it must outperform them on evaluation metrics. The fourth method relies on transformer base models to predict emotions. It is added for comparison with high performing, but bias prone, models to better inform researchers’ decision-making. Each of the methods’ performances is calculated on the test sets of respective datasets.

#### The lexicon approach

The lexicon approach works by averaging the emotional intensity of words in each text. In the case of the Emobank dataset and the Polish political dataset I average the word ratings of previously published transformer-based norm extrapolation models^28^. In the results section I only report the results of averaging after removing stop words, as this method attained better results. As the Emotlas approach has proven superior to the lexicon approaches^11^ in the task of discrete emotion prediction on the GoEmotions dataset, I do not report the performance of the lexicon approach for that specific task.

#### The vader approach

VADER (Valence Aware Dictionary and Sentiment Reasoner) is a rule-based model designed for sentiment analysis, particularly effective in capturing sentiment from social media and informal text. VADER combines a lexicon with rules that account for various intensifiers, negations, and punctuation, making it particularly adept at assessing the sentiment intensity conveyed in short online texts. VADER assigns polarity scores for positive, neutral, and negative sentiment, averaging these scores to produce an overall sentiment value for a given text. This approach is only capable of producing valence estimations^10^.

#### The Emotlas approach

Emotlas utilizes an extensive lexicon-based network to profile emotions by mapping syntactic and semantic relationships in text, effectively capturing nuanced emotional cues without extensive model training. Using validated emotional lexicons for Plutchik’s eight core emotions in conjunction with a spaCy^27^based syntactic analysis, it efficiently identifies emotional tones in multiple languages. Its rule-based structure enables it to run significantly faster than transformer-based models, providing researchers with interpretable insights into how emotions are conveyed in text associations^11^..

#### The transformer approach

A base transformer can be finetuned to predict both continuous and categorical emotions. Here the *RoBERTa -base* transformer model developed by Facebook^31^is finetuned on the two English datasets. After a hyperparameter sweep for each dataset, the final models were trained on the parameter setup that led to the best performance. For the Polish political bias dataset, the performance of the GNN model is compared with a transformer model that was finetuned to predict emotions in the original dataset paper^21^. Debiased transformer-based sentiment models were not included as comparative baselines. Most existing debiasing approaches require the explicit specification of protected attributes or bias dimensions (e.g., gender, race, political orientation) and are therefore designed to mitigate predefined forms of bias^12–14^. In contrast, the central contribution of the present work is a bias-agnostic architectural strategy that does not presuppose which social dimensions are relevant. As such, debiased transformers address a related but conceptually distinct research question. For a more detailed description of the implementation of each of the comparative approaches refer to the Technical Appendix.

### Testing for bias

This section evaluates whether the SProp GNN model mitigates overfitting on biases present in the training data. It follows the approach established by Plisiecki and associates^8^, who analyzed a transformer-based language model’s predictions of sentiment toward 24 prominent Polish politicians (The author thanks Paweł Lenartowicz for his help in devising the statistical bias testing setup for the current study). The original study selected politicians based on a public trust survey and tested the model’s sentiment responses to three types of text stimuli: (1) the politicians’ names alone, (2) politically charged sentences containing these names, and (3) neutral sentences featuring the same names. The model’s task was to classify each stimulus as having positive or negative valence.

To quantify political bias, Plisiecki and associates^8^ fit linear regression models in which the model’s predicted valence was the dependent variable (Y), and the politician’s political affiliation (a dummy-coded factor) and gender were predictors (X1: political affiliation, X2: gender). Thus, the model took the form:

$$\:Y = \beta \:_{0} + \beta \:_{1} \left( {political\:affiliation} \right) + \beta \:_{2} \left( {gender} \right) + \in$$

They found that these predictors explained a substantial proportion of the variance in valence predictions (52% for neutral sentences, 66% for political sentences, and 67% for names alone), indicating significant bias.

The current study replicates and extends this approach with the SProp GNN model using three complementary methods. The goal is to determine whether SProp GNN exhibits significantly less bias than the previous model.

#### Approach 1: replicating the original regression procedure

The first approach directly replicates the original regression methodology. The same linear regression model is applied:

$$\:Y_{\mathrm{SProp}} = \beta \:_{0} + \beta \:_{1} \left( {political\:affiliation} \right) + \beta \:_{2} \left( {gender} \right) + \in$$

Here, $\:{Y}_{\mathrm{SProp}}$ represents the valence predictions made by the SProp GNN for each stimulus. The null hypothesis (H₀) states that the SProp GNN model does not exhibit bias (i.e., *β*_1_ = *β*_2_ = 0) while the alternative hypothesis (H₁) is that at least one of the bias coefficients is non-zero.

To test this, a permutation test on the observed valence predictions is employed. The correspondence between stimuli and the predictor values (political affiliation, gender) is randomly shuffled 100,000 times and the regression is re-estimated each time. This produces a null distribution of test statistics (e.g., F-statistics or sums of squared residuals)^32^. If the observed statistic falls into the extreme tails of this distribution, the H₀ is rejected with the conclusion that the SProp GNN exhibits bias. Non-significant results should be interpreted with caution, as it is difficult to ascertain the test’s power precisely.

#### Approach 2: assessing bias reduction in the SProp model

The second approach aims to determine whether the SProp GNN model reduces bias compared to the original model. Instead of testing if bias exists, the test related to whether the SProp model’s bias is equivalent to (or less than) that of the original transformer model.

First, the SProp GNN predictions are adjusted by removing the estimated bias from the original model. To do this, the original model’s estimated bias coefficients are used ($\:\widehat{{{\upbeta\:}}_{1}}$ and $\:\widehat{{{\upbeta\:}}_{2}}$) to create adjusted predictions:

$$Y_{{\mathrm{SProp,~adjusted}}} = Y_{\mathrm{SProp}} - \left( {\widehat{{\beta _{1} }} \cdot political~affiliation + \widehat{{\beta _{2} }} \cdot gender} \right)$$

Next, a regression is performed with the original bias factors as predictors on these adjusted scores. If the adjusted SProp predictions still show a significant relationship with the bias factors, it means the SProp model retained the same pattern of bias. If the bias factors do not predict the adjusted valence (or predict it inversely), it suggests bias has been reduced.

The null hypothesis (H₀) for this approach states that the SProp model’s bias is the same as the original model’s bias. The alternative hypothesis (H₁) suggests that the SProp model’s bias is reduced. This is tested using a one-sided permutation test (100,000 random assignments). A statistically significant negative beta coefficient would indicate that the SProp model is inversely related to the original bias factor, signifying bias reduction.

#### Approach 3: comparing differences between models

The third approach examines the difference in predictions between the transformer model and the SProp model. Define the difference in predicted valence as:

$$\:{\Delta\:}Y={Y}_{\mathrm{SProp}}-{Y}_{\mathrm{transformer}}$$

This difference is then regressed on the original bias factors:

$$\Delta Y = \gamma _{0} + \gamma _{1} \left( {political~affiliation} \right) + \gamma _{2} \left( {gender} \right) + \in$$

The null hypothesis (H₀) is that the difference in predictions between models is unrelated to the bias factors (γ_1_ = γ_2_ = 0). The alternative hypothesis (H₁) is that these factors significantly predict the difference, confirming that bias is driving the disparities between models.

As before, a one-sided permutation test (100,000 random assignments) is conducted to determine whether the observed association differs from what would be expected by chance. A significant result would indicate that bias plays a key role in differentiating the two models.

#### Note on interpreting the results

While these methods help determine whether the SProp GNN model reduces bias, it is important to recognize that errors in the original model’s estimated bias parameters may attenuate the observed relationships in the SProp model. Due to such estimation errors, the bias parameters in the new tests are not expected to reach exactly 1 (or to show a perfect elimination of bias). Even if no bias were present in the SProp model, random measurement errors and attenuation effects may prevent the parameters from perfectly reflecting the removal of bias.

### Explainability

For the sake of explainability, the SProp GNN saves its scaling factors as well as attention weights, allowing the user to understand the type of information on which the model based its decisions. As a full analysis of how the model reacts to a large array of diverse sentences is beyond the scope of this paper, the focus is shifted to explaining the basic mechanics using two sentences, employing an emotional word and a negation: “I am happy” and “I am not happy.” The activity of the model is then compared between these sentences.

This explainability approach is particularly important because it allows users to assess not only the model’s outputs but also the reasoning behind them. By exposing the scaling factors and attention weights, it becomes possible to pinpoint how specific words and their relationships, such as the negation in “I am not happy,” influence the emotional predictions. This transparency is crucial in ensuring trust and interpretability in emotion prediction models, especially for applications where ethical considerations or fairness are paramount.

## Supplementary Information

Below is the link to the electronic supplementary material.

Supplementary Material 1

## Data Availability

All of the materials available for the present manuscript are available at [https://github.com/hplisiecki/Semantic-Propagation-GNN](), except for the Polish Political Dataset. Because this dataset contains social media posts, the raw texts are not deposited in a public repository due to ethical and legal constraints concerning the sharing of such content. Researchers interested in accessing the dataset for non-commercial scientific purposes may contact the corresponding author directly.

## Code availability

All of the code available for the present manuscript are available at https://github.com/hplisiecki/Semantic-Propagation-GNN.
