# Supplementary material for: Reducing social biases in text-based emotion prediction using semantic blinding and semantic propagation graph neural networks
Source: Sci Rep. 2026 May 8;16:21088. doi: 10.1038/s41598-026-46749-7 (PMC13342623; doi:10.1038/s41598-026-46749-7)
Supplement: Supplementary file 1 — Supplementary Material 1 [file 41598_2026_46749_MOESM1_ESM.docx]

**Technical Appendix**

This technical appendix provides detailed information on the methodologies, models, datasets, and experimental setups used in the paper. It is intended to offer in-depth insights that supplement the main text, as well as to serve as a guide for training similar models in the future.

**Detailed Emotion Prediction Pipeline**

The GNN model proposed in the manuscript consists of three stages:

1. Word level emotion prediction
2. Syntactic graph creation
3. The Semantic Propagation GNN (SProp GNN)

Below each of these stages are described in detail

**Word Level Emotion Prediction**

The model relies on knowing the emotions of every, or most words in a text, to then propagate this information through the syntactic graph and predict emotion on the text level. The paper, in order to predict the emotions of words, draws on the literature in norm extrapolation^1^ which recommends the use of transformer models for word level emotion prediction. The use of these models might not be necessary given a large enough lexicon of words and their respective emotional values, and a corpus with restrained vocabulary. However, to ensure proper word coverage already trained transformer norm extrapolation models are used, or, in the case of discrete emotion prediction, new ones are trained

Transformer based norm extrapolation models are trained by adding a regression or a classification head to a transformer encoder and training it on an existing norm lexicon. Previous research has also added an additional hidden layer between the encoder, and the regression head, with dropout. To create one for the task of discrete emotion prediction the NRC Emotion Intensity Lexicon^2^ was used which provides ratings on a scale of 0 to 1 for 5891 unique words for eight emotions (anger, anticipation, disgust, fear, joy, sadness, surprise, trust). Not all of the words were rated with regards to each emotion, which required training eight separate prediction models for each of the emotions. The "nghuyong/ernie-2.0-base-en" model^3^ was used for each of them, as this was the model that was also used for valence and arousal prediction in a previous paper and thus can be trusted to model emotional information well.^1^ The lexicon was split into seven emotion specific lexicons, and each of these subcorpora was then further divided into train, evaluation, and test sets in the ratio of 8:1:1. Each model was trained for 100 epochs, with a batch size of 500, learning rate of 5e-5, AdamW optimizer with a weight decay of 0.3 and a linear learning rate schedule with warmup steps amounting to 600, a hidden 768 dimensional hidden layer and a dropout of 0.1. Early stopping based on the correlation of predicted scores with the ground truth on the validation set was implemented to prevent overfitting. The test set correlations for each of the emotions are presented in Supplementary Table 1.

| **Supplementary Table 1.** | | | | | | | | |
| --- | --- | --- | --- | --- | --- | --- | --- | --- |
| *Discrete Emotion Norm Extrapolation Model Performance (Pearson’s Correlations)* | | | | | | | | |
| Emotion | Anger | Anticipation | Disgust | Fear | Joy | Sadness | Surprise | Trust |
| Correlation | 0.77*** | 0.68*** | 0.73*** | 0.74*** | 0.71*** | 0.71*** | 0.81*** | 0.72*** |
| * p < 0.6, ** p < 0.1, *** p < 0.001 | | | | | | | | |

The performance metrics of already existing valence and arousal models for Polish and English taken from Plisiecki and Sobieszek^1^ are reported in Supplementary Table 2.

| **Supplementary Table 2.** | | |
| --- | --- | --- |
| *Continuous Norm Extrapolation Model Performance for Polish and English (Pearson’s Correlatrions)* | | |
| Language | English | Polish |
| Valence | 0.95*** | 0.93*** |
| Arousal | 0.76*** | 0.86*** |
| * p < 0.6, ** p < 0.1, *** p < 0.001 | | |

In the pipeline these models were used to assess the emotional value of all words that weren’t stop words, punctuation, or negations, as assessed by the *spaCy* package.^4^ To improve the compute time of the emotion prediction pipeline, using similar models to create a very big lexicon prior to inference can be an option.

**Syntactic Graph Creation**

The current pipeline uses the spaCy package^4^ to split the text into sentences, and words, followed by an analysis of syntactic dependencies. Each word is connected to the other words it relates to syntactically. For example, in the sentence "I do not feel well," spaCy identifies "feel" as the main verb, with "I" as its subject and "well" as its modifier. Additionally, the negation "not" is linked to "feel," indicating a negative sentiment in the phrase. This information can be represented in a graph form where nodes are words, and edges are syntactic dependencies. Each word is furthermore assigned to a specific part-of-speech category (e.g., PRON (pronoun) for "I" and VERB (verb) for "feel") and each dependency labeled accordingly (e.g., nsubj (nominal subject) for "I" as the subject of "feel" and neg (negation) for "not" modifying "feel").

All of punctuation marks are removed from the text, prior to the construction of the graph, apart for the ellipsis, exclamation, and question marks ('…', '! ', '? ') which were retained as they play a big role in the modulation of text meaning. While spaCy recognizes only around 20 part of speech tags, its taxonomy for dependency types is much larger. For this reason, they have been recategorized to a more manageable taxonomy of 15 separate categories with entries like "Descriptive Modifiers of Verbs", or "Negations". The full mapping is available on the paper’s github repository (https://github.com/hplisiecki/Semantic-Propagation-GNN).

The resulting structure is a graph where the nodes (words) are assigned feature vectors with the emotion ratings predicted at the word level emotion prediction stage, along with a number signifying their position in the sentence (word index divided by the number of words in the sentence). Words are also assigned parts of speech indexes signifying their parts of speech categorization. Finally, each of the edges (connections) within the graph gets assigned their dependency indexes, relating to the dependency type taxonomy.

In order to model not only single sentences but also multiple sentences texts, all words from a sentence are additionally to an additional sentence node. When a text has more than one sentence, the sentence nodes relating to each sentence get connected to each other sequentially in the order they appear in text. These sentence nodes are “empty” in the sense that they are not assigned any emotional information. Instead, their emotion node features are initialized at zero, allowing the graph to propagate the emotion from words into them at inference. Their node features also contain their sentence number indicator (sentence index divided by the number of sentences in the text). Finally, they are also assigned a unique parts of speech category (the same for every sentence), with their edges having a unique dependency category (the same for every sentence).

**Semantic Propagation Graph Neural Network**

The SProp GNN rests on the idea of allowing the model to propagate semantic information, in the form of word sentiment scores throughout the syntactic graph as part of the inference. It can do it thanks to the custom SPropConv layer which considers information about the parts of speech each of the two words (nodes) connected in the graph belong to, the emotional information of the receiving node as well as the type of syntactic dependency (edge) between them.

Below is a general overview of the steps that the model performs, followed by a more formal explanation of how the SPropConv layer works, and a short description of the rest of the model’s architecture. Because this paper introduces the SPropConv layer, much attention is paid to its description. Afterwards, the training setup is described.

***General Steps Performed by the SPROP GNN***

1. **Process Syntactic Graph with SPropConv Layer:**

The model processes the syntactic graph of the text using the custom SPropConv layer, enriching each word’s representation with information from related words based on their grammatical structure and roles.

1. **Concatenate with POS Embeddings:**

Each word’s updated features are combined with its part-of-speech (POS) embedding, adding grammatical context to each word’s representation within the graph.

1. **Apply Attention Pooling:**

The concatenated embeddings are passed to an attention pooling layer, which identifies and weighs the most relevant words in the graph for predicting the text’s emotional tone. These weighted embeddings are then aggregated using a global addition pool to create a cohesive text representation.

1. **Pass Through Fully Connected Layers:**

The pooled text representation is further processed through fully connected layers. These layers refine and adjust the representation to reach the dimensionality needed for the final prediction.

1. **Generate Final Prediction:**

For continuous emotional metrics, the output layer uses sigmoid activation to predict values between 0 and 1 for each metric. For discrete emotion categories, a softmax activation generates probabilities across categories, identifying the most likely emotion.

***Mathematical Formulation of the SPropConv Layer***

The SProp layer operates through a series of steps that involve transforming node features, computing messages between nodes, and updating node representations.

**1. Node Feature Transformation**

Each word in the sentence is initially represented by a feature vector which consists of the emotional score of each word, alongside its index sentence divided by sentence length. These features are transformed to a hidden representation using a linear transformation:

$$h_{i}=W_{x}x_{i}+b_{x}$$

- ​$h_{i}$: Hidden representation of node iii.
- ​$W_{x},b_{x}$: Learnable parameters (weights and biases).

**2. Message Passing**

For each edge from node $j$ to node $i$ (representing a syntactic dependency), the model computes a message that incorporates:

- The hidden representation of the source node $h_{j}$
- The embeddings of the POS tags for both nodes: $t_{i}$ for node $\boldsymbol{i}$ and $t_{j}$​ for node $\boldsymbol{j}$.
- The embedding of the edge type (syntactic dependency) $s_{ij}$

These components are concatenated and passed through a linear transformation followed by a hyperbolic tangent activation (tanh) to compute a scaling factor $s_{ij}$

$$\boldsymbol{s}_{\boldsymbol{ij}}\boldsymbol{=}\tanh\left( \boldsymbol{W}_{\boldsymbol{s}}\left[ \boldsymbol{h}_{\boldsymbol{j}}\boldsymbol{;}\boldsymbol{t}_{\boldsymbol{i}}\boldsymbol{;}\boldsymbol{t}_{\boldsymbol{j}}\boldsymbol{;}\boldsymbol{e}_{\boldsymbol{ij}} \right]\boldsymbol{+}\boldsymbol{b}_{\boldsymbol{s}} \right)$$

- $\left[ \boldsymbol{\cdot}\boldsymbol{;}\boldsymbol{\cdot} \right]$ : Concatenation operation.
- $W_{s},b_{s}$: Learnable parameters.

**3. Message Computation**

The message from node $\boldsymbol{j}$ to node $\boldsymbol{i}$ is calculated by scaling the hidden representation of node $\boldsymbol{j}$ with the scaling factor $s_{ij}$​:

$$m_{ij}\boldsymbol{=}\boldsymbol{s}_{\boldsymbol{ij}}\cdot h_{j}$$

This step allows the model to modulate the influence of node $\boldsymbol{j}$ on node $\boldsymbol{i}$ based on their syntactic and semantic relationship.

**4. Aggregation**

For each node $\boldsymbol{i}$, the incoming messages from all its neighboring nodes are aggregated using summation:

$$a_{i}=\sum_{j\in\mathcal{N}\left( i \right)} m_{ij}$$

- $\mathcal{N}\left( i \right)$: Set of neighboring nodes of node iii.

**5. Update**

The node's hidden representation is updated by combining its original hidden state with the aggregated messages, followed by a rectified linear unit (ReLU) activation:

$$h_{i}^{'}=\text{ReLU}\left( h_{i}+a_{i} \right)$$

- $h_{i}^{'}$: Updated hidden representation of node iii.

***The Remaining Architecture***

After the syntactic graph has been processed using the SPropConv layer, the model concatenates the graph’s matrix representation with the parts-of-speech embeddings for each word in the syntactic graph. This concatenated embedding is then passed to an attention pooling layer, which identifies the words in the graph that contain the most relevant information for predicting the text's emotional tone, assigns them weights, and aggregates these embeddings using a global addition pool.

This representation is then passed through fully connected layers that gradually bring them to the dimensionality required by the prediction. In the case of continuous emotional metrics, this means one output dimension per predicted metric, with a sigmoid activation applied to scale the output between 0 and 1. For discrete emotion prediction, the final layer instead uses a softmax activation, outputting probabilities across predefined emotion categories.

***Specific Architectures and Training Setup***

The three SProp GNN models trained on the three datasets GoEmotions, EmoBank, and the Polish Political Dataset share a similar architecture. Each model contains a single SProp layer with 512 hidden dimensions, alongside embedding layers for both parts of speech (node types) and dependency relationships (edge types), with dimensions matching those of the SProp layer. This is followed by a global attention mechanism, which applies a gated attention layer configured with two linear transformations (1024 to 256, and 256 to 1) and a ReLU activation in between. The attention weights are computed by applying softmax across nodes within each graph, and graph-level features are subsequently aggregated using a global addition pool.

The differences between the models lie in the final sequence of linear layers. In the case of discrete predictions, these layers have the form of three linear transformations (1024 to 1024, 1024 to 512, and 512, to the number of discrete emotions), separated by dropout and relu activations. Alternatively, in the case of the two continuous metric prediction models there are only two linear layers (1024 to 100, and 100 to 1), also separated by a dropout and a relu activation. These differences stem from free experimentation with different amounts of final linear layers. A systematic exploration of alternative architectural setups is beyond the scope of this study.

The hyperparameters for the three SProp GNN models were chosen using a Bayesian hyperparameter sweep on the Wandb platform.^5^ The hyperparameter options for the three models were the same: dropout - 0, 0.2, 0.4, 0.6; learning rate - 5e-3, 5e-4, 5e-5, and weight decay - 5e-3, 5e-4, 5e-5. All models were trained using the AdamW optimizer with the epsilon equal to 1e-6 and betas equal to 0.9, and 0.999. The discrete model used the cross-entropy loss, while the continuous metric prediction models used the mean squared error loss. The final models were trained using the best performing parameters from the sweeps.

***Model Size and Compute Time***

To give the reader further intuition about the model size and compute time, the English dimensional model trained on the EmoBank dataset weighs exactly 5.87 MB, and takes exactly 0.1135 seconds to predict emotion for 1000 sentences, with a batch set to 400, and the PC specs: OS: Windows 11 (10.0.26100); CPU: Intel64 Family 6 Model 198 Stepping 2, GenuineIntel; RAM (GB): 64 GB; GPU: NVIDIA GeForce RTX 5080 (15.92 GB VRAM); CUDA: 12.8; Python: 3.12.11; PyTorch: 2.7.1+cu128; PyTorch Geometric: 2.6.1

**Comparative Experiments**

This section will outline the data wrangling performed on the datasets that were used to compare the SProp GNN with other methods, along with the explanation of how each of the alternative methods were implemented.

**Data Wrangling**

Each of the datasets was processed for the task of using them to compare alternative approaches to emotion prediction. Considerable attention was paid to the description of the Polish political dataset as it is a far less known dataset when compared to the other two.

***The Goemotions Dataset***

The goemotions dataset was developed by a team at Google.^6^ It consists of 57565 unique texts and 210622 annotations. Each comment received annotations from three English-speaking raters from India, with additional raters assigned when agreement was low. The most voted for emotion per each text was computed and those texts for which two emotions were assigned the same number of votes were dropped. This resulted in a dataset of 47136 unique texts. From these texts, those that were assigned one of the following emotions: anger, disgust, fear, joy, surprise; were retained leaving 4819 unique texts. The choice of emotions was dictated by the availability of emotion norms in the NRC Emotion Intensity Lexicon.^2^ This dataset was then split into the training, evaluation, and test sets in the proportion of 8:1:1.

***The EmoBank Dataset***

The EmoBank dataset, created by Buechel and Hahn,^7^ consists of 10,062 English sentences from sources like news, blogs, fiction, and letters, annotated along three emotional dimensions: Valence, Arousal, and Dominance (VAD). Each sentence was rated by multiple annotators from the crowdsourcing platform CrowdFlower for both *writer* and *reader* perspectives, giving insights into both expressed and perceived emotions. Each sentence was annotated by 5 annotators. In accordance with the recommendations of the researchers, the dataset with the weighted average of the reader and writer perspective labels provided at their online repository was used for training.^8^ The ratings for valence and arousal were normalized to a 0 to 1 range by subtracting the lowest score and dividing by the number of Likert scoring options prior to splitting into the training, evaluation, and test sets in the proportion of 8:1:1.

***The Polish Political Dataset***

The Polish Political dataset^9^ was created by sampling text data from social media profiles of Polish journalists, politicians, and non-governmental organizations (NGOs) across YouTube, Twitter, and Facebook. Posts from 2019 onward were collected for 69 profiles. A total of 1,246,337 text snippets were gathered, with breakdowns of 789,490 tweets, 42,252 YouTube comments, and 414,595 Facebook posts. To handle the varying text lengths, Facebook posts were split into sentences, and only texts under 280 characters were retained. Social media artifacts, such as dates and extraneous links, were removed, and non-Polish texts were filtered using language detection software. To prevent overfitting, online links and usernames were standardized as "*link*" and "*user*."

To create a dataset with richer emotional content, neutral texts were filtered out, leaving only those with higher levels of emotional valence, arousal, and dominance. This selection process used a lexicon-based approach, where each text was assessed for emotional intensity across these dimensions, resulting in 8,000 emotionally charged texts. An additional 2,000 neutral texts were included to balance the dataset, preserving original platform proportions. The final 10,000-text dataset, comprising 496 YouTube comments, 6,105 tweets, and 3,399 Facebook texts, was then annotated by 20 psychology students well-versed in Polish political discourse. Each text was rated by five randomly assigned annotators on six emotions (happiness, sadness, disgust, fear, anger, and pride) and two emotional dimensions (valence and arousal), using a 5-point Likert scale. Before formal annotation, annotators received an introduction to valence and arousal, and comprehensive guidelines were provided to ensure consistency. For clarity, the annotators received the following English instruction for evaluating valence and arousal:

"Go back to the text you just read. Now think about the sign of emotion (positive / negative) and the arousal you read in a given text (no arousal / extreme arousal). Rate the text on these emotional dimensions."

This instruction was designed to provide a standardized understanding of emotional dimensions, ensuring alignment in annotators' assessments across the dataset.

For the purposes of the current experiment, all of the emoticons and symbols were filtered out and the dataset was split into the training, evaluation, and test sets in 8:1:1 proportion.

**Comparative Approaches**

This section outlines the details of how each of the alternative approaches was set up and trained for performance comparison.

***The Lexicon Approach***

For the lexicon analysis of the EmoBank, and the Polish Political dataset I utilize the norm extrapolation transformer-based models for Polish and English described in the “Word Level Emotion Prediction” section above. Each test set text was first split into words using the *spacy* package.^4^ Each word that wasn’t a stop word was then fed into the norm extrapolation model, and the emotional prediction was averaged to get the text level emotion score.

***The Vader Approach***

VADER (Valence Aware Dictionary and Sentiment Reasoner) is a rule-based model designed for sentiment analysis, particularly effective in capturing sentiment from social media and informal text. I have used the 3.3.2 version of the Vader package to get the valence/positivity scores for the Emobank test set.

***The EmoAtlas Approach***

The EmoAtlas utilizes an extensive lexicon-based network to profile emotions by mapping syntactic and semantic relationships in text, effectively capturing nuanced emotional cues without extensive model training. The EmoAtlas performance results were computed using its official implementation on the GoEmotions test set.

***The Transformer Approach***

For the GoEmotions and EmoBank datasets the *roberta-base* transformer model developed by Facebook was finetuned on the two English datasets.^11^ A fully connected layer, with the dimensions equal to 768 was added on the top of the base model with dropout and a layer norm, with either a regression head for the sake of predicting valence and arousal, or a classification head for predicting discrete emotions. A Bayesian hyperparameter sweep was performed using the Wandb platform^5^ for both models with 20 runs and the following hyperparameter options: dropout – 0.0, 0.2, 0.4, 0.6; learning rate – 5e-4, 5e-5, 5e-6; weight decay – 0.0, 0.2, 0.4, 0.6; and warmup steps – 300, 600, 900. Both models use the AdamW optimizer for training with the epsilon equal to 1e-6 and betas equal to 0.9, and 0.999, alongside the linear learning rate scheduler with warmup. In the case of discrete prediction, cross-entropy loss was used, while in the case of continuous emotion metric prediction mean squared error loss was chosen. Finally, the final models have been trained using the best performing hyperparameters from the sweep. The performance of each of the models is reported in the results section of the main manuscript. The training code for these models can be found in the following Google Collab:
https://colab.research.google.com/drive/1pA3oBbHg0pza1yF5kyuddK36-RGKtHxo?usp=sharing

**SProp GNN Pseudocode**

The following pseudocode summarizes the computational steps performed by the Semantic Propagation Graph Neural Network (SProp GNN) during a single forward pass. It provides a conceptual, implementation-agnostic overview of how syntactic structure and token-level semantic cues are combined, propagated through the dependency graph, and aggregated to produce a sentence-level prediction.

| PSEUDOCODE: SProp GNN with Norm-Extrapolation Token Cues  INPUT:  Text input T  Dependency parser (spaCy)  NormExtrap model f_NE(·) # word-level emotion prediction model  Stopword list SW  Learned parameters:  E_pos[·] : POS / node-type embedding lookup  E_dep[·] : dependency-relation embedding lookup  SPropLayer : semantic propagation (message passing) layer  AttnPool : attention pooling module  Head : output head (regression/classification)  OUTPUT:  y_hat : sentence-level prediction  {alpha_i} : node attention weights (optional, for visualization)  {s_ij} : edge scaling factors (optional, for visualization)  ------------------------------------------------------------ STEP 1: CONSTRUCT SYNTACTIC GRAPH FROM spaCy ------------------------------------------------------------ doc = spacy_parse(T)  Create node v_i for each token t_i in doc Create directed edges e_ij for dependency arcs (head -> dependent) Store edge type r_ij for each edge (dependency relation label)  ------------------------------------------------------------ STEP 2: COMPUTE TOKEN-LEVEL EMOTION CUES USING NORM EXTRAPOLATION ------------------------------------------------------------ For each token node v_i corresponding to token t_i:  If t_i in SW:  cue_i = ZERO_VECTOR # or omit/neutralize cue for stopwords  Else:  cue_i = f_NE(t_i) # norm-extrapolated word-level emotion cue(s)   pos_i = POS_tag(t_i)  x_i = CONCAT( cue_i , E_pos[pos_i] ) # initial node feature vector  h_i(0) = x_i  For each directed edge e_ij:  r_ij = dependency_type(e_ij)  e_ij_embed = E_dep[r_ij] # edge-type embedding  ------------------------------------------------------------ STEP 3: SEMANTIC PROPAGATION (MESSAGE PASSING OVER DEPENDENCIES) ------------------------------------------------------------ For layer l = 1 ... L:   For each directed edge (i -> j) with edge type r_ij:  s_ij(l) = ScalingNetwork( e_ij_embed ) # relation-conditioned scaling/gate  m_ij(l) = s_ij(l) ⊙ Transform( h_i(l-1) ) # message from i to j (scaled elementwise)   For each node j:  agg_j(l) = AGGREGATE( m_ij(l) over all incoming edges i -> j )  h_j(l) = UPDATE( h_j(l-1), agg_j(l) ) # e.g., residual + nonlinearity (+ norm)  Let H = { h_i(L) } be final node representations  ------------------------------------------------------------ STEP 4: ATTENTION POOLING (GRAPH -> SENTENCE VECTOR) ------------------------------------------------------------ For each node i:  a_i = AttnScore( h_i(L) ) # scalar logit alpha_i = softmax(a_i over nodes i) g = SUM_i [ alpha_i * h_i(L) ] # sentence representation  ------------------------------------------------------------ STEP 5: OUTPUT PREDICTION ------------------------------------------------------------ y_hat = Head(g)  Return y_hat, {alpha_i}, {s_ij} |
| --- |

**References**

1. Plisiecki, H., & Sobieszek, A. (2023). Extrapolation of affective norms using transformer-based neural networks and its application to experimental stimuli selection. *Behavior Research Methods*, *56*(5), 4716–4731. <https://doi.org/10.3758/s13428-023-02212-3>

2. Mohammad, S. M. (2017). Word Affect Intensities (Version 2). arXiv. <https://doi.org/10.48550/ARXIV.1704.08798>

3. Sun, Y., Wang, S., Li, Y., Feng, S., Tian, H., Wu, H., & Wang, H. (2019). *ERNIE 2.0: A continual pre‑training framework for language understanding* [Preprint]. *arXiv*. <http://arxiv.org/abs/1907.12412>

4. Ines Montani, Matthew Honnibal, Adriane Boyd, Sofie Van Landeghem, & Henning Peters. (2023). *explosion/spaCy: V3.7.2: Fixes for APIs and requirements* (Version v3.7.2) [Computer software]. Zenodo. <https://doi.org/10.5281/ZENODO.1212303>

5. *Wandb/wandb*. (2024). [Python]. Weights & Biases. <https://github.com/wandb/wandb> (Original work published 2017)

6. Demszky, D., Movshovitz-Attias, D., Ko, J., Cowen, A., Nemade, G., & Ravi, S. (2020). *GoEmotions: A Dataset of Fine-Grained Emotions* (No. arXiv:2005.00547). arXiv. <https://doi.org/10.48550/arXiv.2005.00547>

7. Buechel, S., & Hahn, U. (2022). *EmoBank: Studying the Impact of Annotation Perspective and Representation Format on Dimensional Emotion Analysis* (Version 1). arXiv. <https://doi.org/10.48550/ARXIV.2205.01996>

8. Buechel, S., & Hahn, U. (2022). *EmoBank* [Dataset]. <https://github.com/JULIELab/EmoBank>

9. Plisiecki, H., Koc, P., Flakus, M., & Pokropek, A. (2024). *Predicting Emotion Intensity in Polish Political Texts: Comparing Supervised Models and Large Language Models in a Resource-Poor Language* (Version 1). arXiv. <https://doi.org/10.48550/ARXIV.2407.12141>

10. Semeraro, A., Vilella, S., Mohammad, S., Ruffo, G., & Stella, M. (2023). *EmoAtlas: An emotional profiling tool merging psychological lexicons, artificial intelligence and network science*. <https://doi.org/10.21203/rs.3.rs-2428155/v1>

11. Liu, Y., Ott, M., Goyal, N., Du, J., Joshi, M., Chen, D., Levy, O., Lewis, M., Zettlemoyer, L., & Stoyanov, V. (2019). *RoBERTa: A Robustly Optimized BERT Pretraining Approach* (No. arXiv:1907.11692). arXiv. <http://arxiv.org/abs/1907.11692>
